# Evaluation of T2-W MR imaging and diffusion-weighted imaging for the early post-treatment local response assessment of patients treated conservatively for cervical cancer: a multicentre study

**DOI:** 10.1007/s00330-018-5510-3

**Published:** 2018-06-25

**Authors:** Maarten G. Thomeer, Vincent Vandecaveye, Loes Braun, Frenchey Mayer, Martine Franckena-Schouten, Peter de Boer, Jaap Stoker, Erik Van Limbergen, Marrije Buist, Ignace Vergote, Myriam Hunink, Helena van Doorn

**Affiliations:** 1 Department of Radiology and Nuclear Medicine, Erasmus University Medical Center, Rotterdam, The Netherlands; 2 Department of Radiology, Erasmus Medical Center Rotterdam, P.O Box 2040, 3015 CE Rotterdam, The Netherlands; 3 Department of Radiology, University Hospitals Leuven, Leuven, Belgium; 4 Department of Gynecology, Erasmus University Medical Center, Rotterdam, The Netherlands; 5 Department of Radiotherapy, Erasmus University Medical Center, Rotterdam, The Netherlands; 6 Department of Radiotherapy, Academic Medical Center, University of Amsterdam, Amsterdam, The Netherlands; 7 Department of Radiology and Nuclear Medicine, Academic Medical Center, University of Amsterdam, Amsterdam, The Netherlands; 8 Department of Radiotherapy, University Hospitals Leuven, Leuven, Belgium; 9 Department of Gynecology Academic Medical Center, University of Amsterdam, Amsterdam, The Netherlands; 10 Department of Gynecology, University Hospitals Leuven, Leuven, Belgium; 11 Department of Epidemiology, Erasmus University Medical Center, Rotterdam, The Netherlands

**Keywords:** Uterine cervical neoplasm, Magnetic resonance imaging, Diffusion-weighted magnetic resonance imaging, Radiation, Comparative study

## Abstract

**Objectives:**

To compare MR imaging with or without DWI and clinical response evaluation (CRE) in the local control evaluation of cervical carcinoma after radiotherapy.

**Methods:**

In a multicentre university setting, we prospectively included 107 patients with primary cervical cancer treated with radiotherapy. Sensitivity and specificity for CRE and MR imaging (with pre-therapy MR imaging as reference) (2 readers) were evaluated using cautious and strict criteria for identifying residual tumour. Nested logistic regression models were constructed for CRE, subsequently adding MR imaging with and without DWI as independent variables, as well as the pre- to post-treatment change in apparent diffusion coefficient (delta ADC).

**Results:**

Using cautious criteria, CRE and MR imaging with DWI (reader 1/reader 2) have comparable high specificity (83% and 89%/95%, respectively), whereas MR imaging without DWI showed significantly lower specificity (63%/53%) than CRE. Using strict criteria, CRE and MR imaging with DWI both showed very high specificity (99% and 92%/95%, respectively), whereas MR imaging without DWI showed significantly lower specificity (89%/77%) than CRE. All sensitivities were not significantly different. Addition of MR imaging with DWI to CRE has statistically significant incremental value in identifying residual tumour (reader 1: estimate, 1.06; *p* = 0.001) (reader 2: estimate, 0.62; *p* = 0.02). Adding the delta ADC did not have significant incremental value in detecting residual tumour.

**Conclusions:**

DWI significantly increases the specificity of MR imaging in the detection of local residual tumour. Furthermore, MR imaging with DWI has significant incremental diagnostic value over CRE, whereas adding the delta ADC has no incremental diagnostic value.

**Key Points:**

*• If MR imaging is used for response evaluation, DWI should be incorporated*

*• MR imaging with DWI has diagnostic value comparable/complementary to clinical response evaluation*

*• Inter-reader agreement is moderate to fair for two experienced radiologist readers*

*• Quantitative measurements of ADC early post-therapy have limited diagnostic value*

## Introduction

Cervical cancer represents a major health burden, with for instance around 12,000 newly diagnosed patients in the USA each year (US Cancer Statistics Working Group 2016). The standard treatment for locally advanced cervical cancer is external beam radiotherapy with image-guided adaptive brachytherapy [1, 2] and concurrent chemotherapy [3, 4] or hyperthermia [5]. More than 90% of patients show complete local response after such treatment [6–8].

To detect local residual tumour at an early time point (2–3 months after treatment) and allow potentially curative salvage therapy, response assessment after radiotherapy is uniformly recommended [9–11]. Although the effect of salvage surgery on survival is not well studied, retrospective studies suggest that early detection of local residual disease in asymptomatic women may offer survival benefit [12, 13]. Moreover, salvage hysterectomy for local disease at a later, i.e. symptomatic, stage has high complication rates or is not feasible because of irresectable disease [14]. Therefore, a well-founded diagnosis of local residual tumour shortly after completing radiotherapy would be helpful in the timely selection of women for salvage surgery, and might contribute to enhanced survival. Secondly, the patient with residual disease not suitable for salvage surgery might be quickly informed about her unfortunate prognosis in a timely manner.

In most centres, response assessment is performed via clinical response examination (CRE) at 2–3 months after radiotherapy [1, 2]. This pelvic exam can be unsatisfactory because of the altered anatomy after radiotherapy (no cervix, stricture of the vagina, fibrosis) and the discomfort and pain experienced by patients. Subsequently an examination under anaesthesia may be necessary.

A seemingly attractive alternative after completing radiotherapy therefore is imaging, either with PET CT scanning or magnetic resonance (MR) imaging.

The use of PET-CT has recently been advised for response evaluation according to the National Comprehensive Cancer Network (NCCN 2017). In the earlier European Society of Medical Oncology guideline PET-CT is not incorporated for response evaluation [15]. As far as we know, no data exists on its diagnostic value in the first response evaluation. For this reason we do not use PET-CT in our departments. Further prospective studies are needed to ensure its diagnostic value.

MR imaging has so far been unable to fulfil the need to detect residual disease after radiation because of a false positive rate of up to 45% [16, 17] that is mainly attributable to difficulties in differentiating residual tumour from local oedema. The development of MR imaging with diffusion-weighted imaging (DWI) has led to investigations of its potential in differentiating high cellular matrix (as seen in tumour) from low cellular matrix (as seen in oedema). Several studies have shown promising results in identifying local residual tumour after radiotherapy, for instance in rectal cancer and breast cancer [18–21]. In 2015, a systematic review described the additional value of DWI in monitoring treatment response in patients undergoing chemoradiotherapy for locally advanced uterine cervical cancer [21]. A greater increase of the apparent diffusion coefficient (ADC) was seen after treatment versus before treatment if response was complete. However, data were not externally validated. Moreover, reference standard was mostly based on MR imaging findings. To the best of our knowledge, only two recent studies have evaluated the accuracy of MR imaging with DWI [22, 23]. The first study was a single-centre study of retrospective design, while the second study involved retrospective analysis without standard use of pathologic evidence as a reference standard, instead also using radiological follow-up.

The primary aim of our study was to perform a multicentre study of the incremental value of MR imaging with DWI compared to standard MR imaging in the first response evaluation after radiotherapy for cervical cancer. A secondary aim was to evaluate the additional value of MR imaging with or without DWI in comparison to CRE. Histopathology or a minimal follow-up of 1 year was used as reference standard.

## Materials and methods

### Study design and patients

This prospective study was approved by the institutional review board in the three participating university hospitals (Erasmus University Medical Centre Rotterdam, Academic Medical Centre Amsterdam, University Hospitals Leuven) and written informed consent was obtained from all patients. The study was registered under trial ID (NTR3345). Study reporting was in accordance with the Standards for Reporting Diagnostic Accuracy Studies (STARD 2015).

From March 2011 to April 2015, all patients of 18 years and older with a primary tumour of the cervix and scheduled for radiation therapy with curative intent were included. Pregnant women, women with a contraindication for general anaesthesia or MRI and women with cognitive deficits were excluded.

### Clinical response examination

CRE was based on a pelvic exam, performed by a staff or fellow gynaecological oncologist. CRE included visualisation of the cervix and vagina with a speculum and bimanual rectovaginal palpation.

Treatment response was scored prospectively as either suspicious or not suspicious for residual tumour and was labelled ‘indeterminate’ when patients were too difficult to evaluate, when findings were equivocal, or when the patient refused evaluation at the outpatient clinic. The reader extracting the indeterminate results retrospectively from the medical records was blinded to the MR imaging findings.

For CRE, sensitivity and specificity were calculated on the basis of cautious criteria (i.e. indeterminate results were classified as residual tumour) and on strict criteria (i.e. indeterminate results were classified as complete response).

As a result of ethics requirements at one centre, the T2-W MRI alone was available to the treating clinicians after CRE. In no patient was the information from DWI used. Where CRE was negative and MR imaging findings without DWI were suspicious for residual tumour, a subsequent clinical examination under anaesthesia was performed or a closer follow-up proposed. Although this could induce verification bias in some cases, it probably had no influence on the reference standard result, since residual tumour would only be detected earlier than during follow-up. In no patient was the information of DWI used.

### MR imaging

In each participating centre, at a predefined time point around 2 months after treatment, imaging was performed on a state-of-the-art 3.0-Tesla MR scanner. In each participating centre, pre-treatment MR imaging was done within 4 weeks of treatment start and response MR imaging was planned 6–8 weeks after finalising radiotherapy. The protocol differed slightly between different vendors (Philips, Best, the Netherlands and General Electrics, Milwaukee, WI). Protocols can be found in Table 1. Imaging was performed before start of treatment and after completing radiotherapy. One institution used intravaginal gel.

**Table 1:** The protocol differed slightly between the different vendors (Philips Healthcare, Best, the Netherlands and General Electrics, Milwaukee, Wis). Imaging was performed before the start of treatment and after completing radiotherapy. One institution used intravaginal gel (Fig. 2b)

Scanner (3 Tesla)		GE scanner (centre 1)	Philips scanner (centres 2 and 3)
Patients included		85	22
T2-weighting (2D)	TR/TE (ms/ms)	9436/81	4571/80
	Scan plane	3 directions	3 directions
	Flip angle (grades)	120	90
	Matrix size	320 × 320	344× 285
	Bandwidth (kHz)	125	125
	Field view	24	24
	Number of excitations	2	1
	Slice thickness (mm)	4	4
	Gap	0.4	0.4
DWI (2D)	Gradient	3 directions	3 directions
	Scan plane	Axial	Axial
	*b* values	0–50–100–500–750–1000	0–50–100–500–750–1000
	Bandwidth (kHz)	125	125
	Slice thickness (mm)	4	4

### MR image analysis

MR imaging was reviewed independently by two experienced readers (reader 1, an abdominal radiologist for more than 14 years; reader 2, an abdominal radiologist for more than 10 years). Both readers were blinded to clinical and pathological results. The pre-therapy images were at the readers’ disposal to correlate the location of the primary tumour, similar to the evaluation process performed in daily clinical practice.

Evaluation was based on first (1) T2-weighted MR images (T2-WI) in three directions, then (2) T2-WI with DWI, and finally (3) the change in apparent diffusion coefficient (δADC) between post-therapy and pre-therapy.

The post-therapy DWI was analysed first together with the information from before treatment. This is very helpful for localisation. The rationale for this was to imitate clinical routine as much as possible. We assume that in clinical practice all patients have an initial scan before therapy.

The criterion for residual tumour based on T2-WI was a solid residual mass with intermediate signal intensity. The criterion for residual tumour based on DWI was b1000 hyperintensity, not attributable to T2 shine-through, at the location of the primary tumour as correlated to the T2-WI. For the DWI evaluation, qualitative interpretation of the ADC map provided additional information when deemed necessary by the reader. For example, if the lesion had high intensity on DWI with a corresponding low value on the ADC map (based on qualitative interpretation without quantitative measurement), it was interpreted as tumour. In the third evaluation, based on objective quantitative measurement of the ADC values, the criterion for residual tumour was δADC ≤ 31.0 × 10^−4^. As published earlier, a δADC ≥ 62.0 × 10^−4^ was considered a complete response [21].

The ADC was measured by circular region of interest (ROI) as large as possible within the solid tumour on the ADC maps on the largest axial tumour slice. Special attention was paid to avoid the areas of focal signal intensity changes, susceptibility artefacts and necrosis. If during response evaluation no tumour was visible, the location of the tumour on the pre-therapy scan was used for ROI measurements. While the justification for this approach is debatable, in most previous studies on cervical carcinoma quantification has been performed in the same way, making it at least comparable [21].

The radiological response of the primary tumour was scored using a five-point score (1 = definite complete response, 2 = probable complete response, 3 = indeterminate result, 4 = probable residual tumour, 5 = definite residual tumour). As for CRE, sensitivity and specificity were calculated on the basis of cautious criteria (i.e. indeterminate results were classified as residual tumour) and on strict criteria (i.e. indeterminate results were classified as complete response).

### Reference standard

All patients underwent a pelvic examination after radiotherapy and subsequently every 3 months, for at least a year. It is assumed that most small, clinically overt, residual tumours would become obvious within this follow-up period, although some recurrences can occur up to 3 years after therapy. Histopathological evaluation served as the reference standard in case of positive results. A recurrence-free follow-up period of at least 1 year was considered a surrogate reference standard for a complete local response. Routine histopathology in all cases would have added the burden of an additional examination under anaesthetic (EUA) and was deemed unacceptable.

### Statistical analysis

For interpretation of T2-weighted images with or without DWI, interobserver agreement on the five-point scale was calculated via Cohen’s kappa, using a weighted method based on the squared distance from the diagonal. Kappa values of 0–0.20 were interpreted as poor agreement, 0.21–0.40 as fair, 0.41–0.60 as moderate, 0.61–0.80 as good and 0.81–1 as excellent agreement.

A logistic regression model was constructed first with CRE only (model 1), and subsequently by adding T2-WI (model 2), T2-WI with DWI (model 3) and δADC (model 4) as independent variables for identification of residual tumour (dependent variable) by the two radiology readers. Our sample size calculation was based on optimising specificity, in order to decrease the false positive rate due to local oedema. We chose an alpha value of 0.05 and estimated the prevalence of residual tumour at 10%. To demonstrate a significant difference in the expected specificity of 0.70 compared to the previously found specificity of 0.55, with a power of 0.80, would require 83 patients without residual tumour. With 10% expected to have residual tumour and 90% a complete response, 92 patients were required. Allowing for 10% loss to follow-up would mean that we needed 101 patients.

## Results

### Patient and response characteristics

A total of 133 patients were eligible; 26 patients were excluded [refusal of further inclusion (*n* = 17), change of therapy (*n* = 5), further therapy elsewhere (*n* = 2), death during therapy (*n* = 1), claustrophobia in MR scanner (*n* = 1)], leaving 107 patients for analyses. Baseline demographics and clinical characteristics are shown in Table 2. The reason for the conservative treatment of the patient with a FIGO Ia was a relative contraindication for operation.

**Table 2:** Patient characteristics

Patient characteristics		
Total number of patients	107	
Age (median, range)	49 (27–84)	
Tumour stage		
FIGO	Number	%
IA	1	1
IB1	4	4
IB2	13	12
IIA	5	5
IIB	67	63
IIIA	3	3
IIIB	10	9
IVA	4	4
Histologic subtype		
Squamous cell carcinoma	87	81
Adenocarcinoma	13	12
Other	7	7
Treatment		
Chemoradiation	80	75
Radiotherapy + hyperthermia	27	25
(± Induction chemotherapy)		
Brachytherapy	105	98
No brachytherapy	2	2

**Fig. 1:**
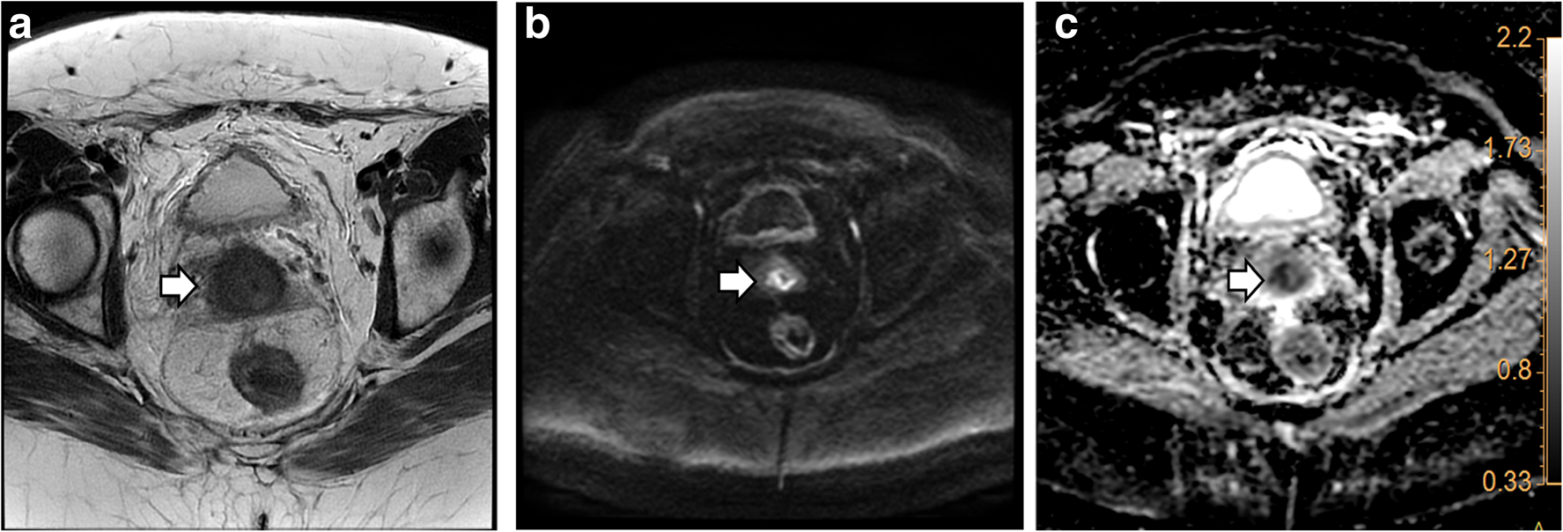
This 50-year-old patient was clinically evaluated as non-suspect 61 days after radiation therapy. T2-weighted imaging (**a**, arrow) showed an indeterminate aspect of the cervix and was therefore supplemented with true cut biopsy under anaesthesia. This confirmed residual disease on histopathology. At the end of the study diffusion-weighted imaging (DWI) was analysed, blinded for the histopathology result. This showed (**b**, arrow) a clear hyperintense region. Visual ADC information (low intensity) was used to differentiate from mucosal oedema (**c**, arrow), leading to the diagnosis of residual tumour by both readers. The δADC was only suspect for local residue for reader 2 (δADC, reader 1: 52 × 10^−4^ and reader 2: 29 × 10^−4^) on the basis of aforementioned cut-off of 31 × 10^−4^

**Fig. 2:**
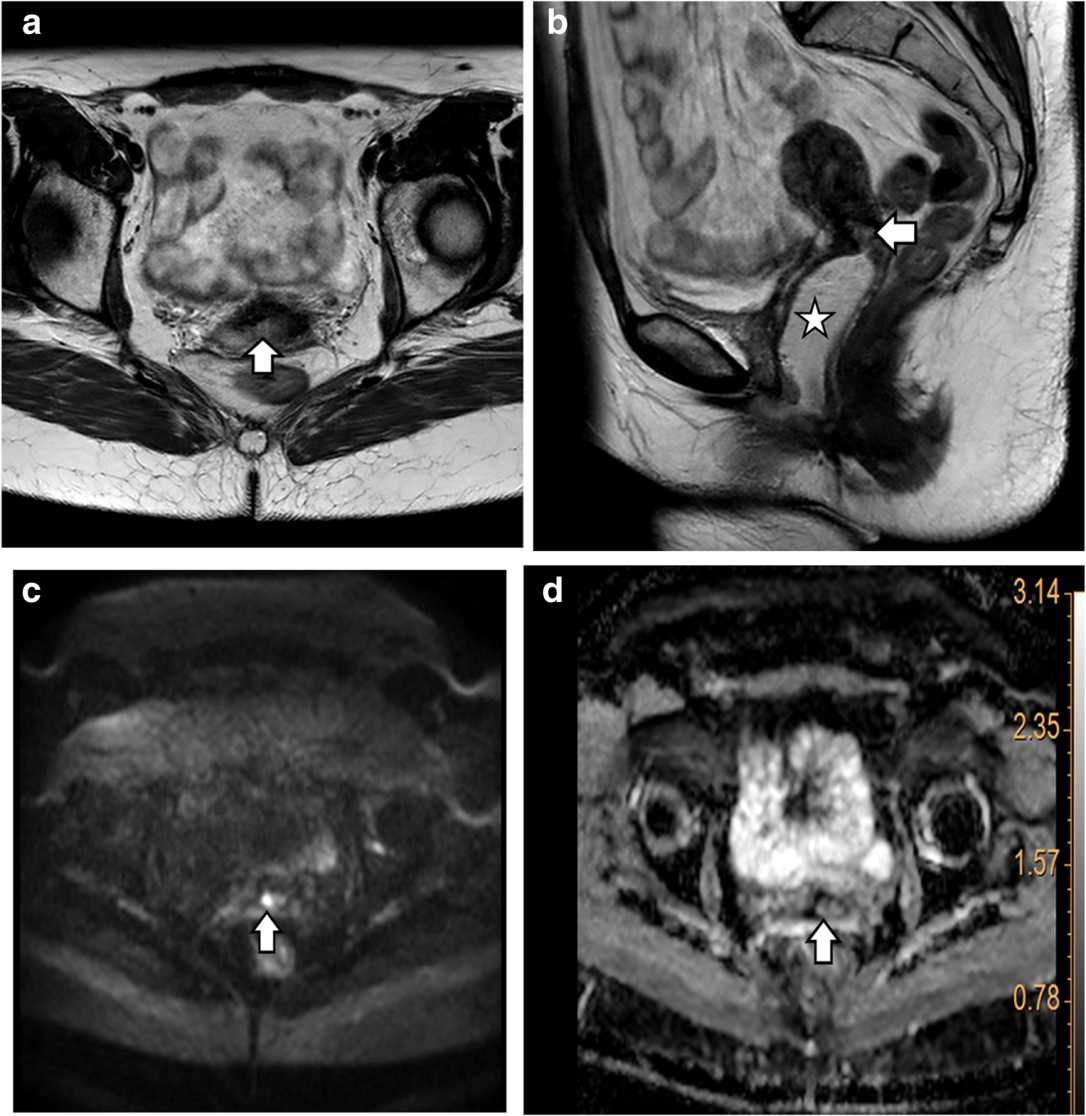
A 30-year-old patient with a normal post-therapeutic clinical examination. T2-weighted MR imaging (**a**, **b** arrow) showed an infracentimetric suspicious region centrally in the cervix. Local follow-up during 1 year remained normal. Diffusion-weighted imaging (DWI, **c** arrow) was not suspect for both readers. The ADC map (**d** arrow) was hyperintense (ADC, reader 1: 132 × 10^−4^ mm^2^/s and reader 2: 125 × 10^−4^ mm^2^/s), confirming the presence of fluid rather than tumour. The delta ADC was not suspect for readers (δADC, reader 1: 63 × 10^−4^ and reader 2: 52 × 10^−4^) on the basis of aforementioned cut-off of 31 × 10^−4^. Note the use of intravaginal gel in order to expand the vagina on **b** (white star)

A complete response was found in 95 patients, whereas 12 patients (11%) had local residual tumour on biopsy or resection (see examples in Figs. 1 and 2). The range of the tumour maximal diameter was between 1 mm and 62 mm, with a mean of 24 mm and a standard deviation of 21 mm. The mean time interval between the end of radiotherapy and response evaluation was 51 days (95% CI 48–53) for CRE and 42 days (95% CI 40–44) for MRI. Follow-up duration in patients without recurrence was between 1.1 and 5.2 years.

### Interobserver agreement

Interobserver agreement on a five-point scale was fair (0.32) for interpretation of T2-WI and moderate (0.46) for the interpretation of T2-WI + DWI. When the five-point scale was converted to a three-point scale, interobserver agreement was fair for interpretation of T2-WI (0.26) and T2-WI + DWI (0.39).

### Diagnostic performance of CRE and MR imaging

Diagnostic performance in the interpretation of CRE, T2-WI and T2-WI + DWI are shown in Table 3. Almost all specificities of T2-WI with DWI (asterisk) were significantly higher compared to T2-WI. The sensitivities of T2-WI with DWI did not differ significantly compared to T2-WI.

**Table 3:** CRE and MR imaging findings of local residual tumour with corresponding diagnostic values using cautious and strict criteria. Using cautious criteria indeterminate findings were labeled as suspicious for residual tumour and using strict criteria indeterminate findings were labeled as not suspicious for residual tumour. CRE clinical response examination, T2-WI T2-weighted MR imaging, T2-WI + DWI T2-weighted imaging + diffusion-weighted imaging, CI 95% confidence interval. Asterisk refers to the outcome which was significantly different on T2-WI + DWI compared to T2-WI. Bold highlights figures which were significantly different on T2-WI or T2-WI + DWI compared to CRE

		Cautions criteria		Strict criteria	
		Sensitivity	Specificity	Sensitivity	Specificity
CRE		58% (7/12)	83% (79/95)	25% (3/12)	99% (94/95)
		(CI 32–82)	(CI 76–89)	(CI 7–53)	(CI 95–100)
T2-WI	Reader 1	67% (8/12)	**63% (60/95)**	58% (7/12)	**89% (85/95)**
		(CI 39–88)	**(CI 54–71)**	(CI 32–82)	**(CI 83–94)**
	Reader 2	**83% (10/12)**	**53% (50/95)**	75% (9/12)	**77% (73/95)**
		**(CI 56–97)**	**(CI 44–61)**	(CI 47–93)	**(CI 69–84)**
T2-WI+DWI	Reader 1	**83% (10/12)**	89% (85/95)*	**83% (10/12)**	92% (87/95)
		**(CI 56–97)**	(CI 83–94)	**(CI 56–97)**	(CI 85–96)
	Reader 2	50% (6/12)	95% (90/95)*	50% (6/12)	95% (90/95)*
		(CI 25–75)	(CI 89–98)	(CI 25–75)	(CI 89–98)

*T2-WI with DWI statistically significantly higher compared to T2-WI

Compared to CRE, MR imaging had significantly lower specificities in all situations (bold), and sensitivities and specificities of T2-WI with DWI were not significantly different. Only for reader 1 in the strict setting was sensitivity significantly higher than that for CRE (bold).

Logistic regression analyses of the effect of adding different MR imaging protocols to the CRE are shown in Table 4. The model with only CRE (model 1) demonstrated the value of CRE (β = 1.74, *p* = 0.0006) in identifying residual tumour. Model 2 demonstrated the significant added value of T2-WI in addition to CRE (reader 1: β = 0.77, *p* = 0.009) (reader 2: β = 0.88, *p* = 0.01). Adding DWI to the T2-WI protocol (model 3) demonstrated the significant added value of DWI (reader 1: β = 1.06, *p* = 0.001) (reader 2: β = 0.62, *p* = 0.02) and showed that T2-WI with DWI can replace CRE. The addition of the δADC (model 4) had no added value for either reader.

**Table 4:** Logistic regression analyses of addition of various MR imaging protocols to the CRE. Parameters with significant p values are shown in bold. CRE clinical response examination, T2-WI T2-weighted MR imaging, T2-WI + DWI T2-weighted imaging + diffusion-weighted imaging, δADC is the difference between the pretreatment ADC and the post-treatment ADC,ADC apparent diffusion coefficient, estim. estimate, std err. standard error

Reader 1				Reader 2			
**Model 1**				**Model 1**			
	Estim.	Std. err.	*p*-value		Estim.	Std. err.	*p*-value
Intercept	-2.84	0.46	*p*<0.0001	Intercept	-2.84	0.46	*p*<0.0001
CRE	1.74	0.50	**0.0006**	CRE	1.74	0.51	**0.0006**
**Model 2**				**Model 2**			
	Estim.	Std. err.	*p*-value		Estim.	Std. err.	*p*-value
Intercept	-4.61	0.95	*p*<0.0001	Intercept	-5.52	1.30	*p*<0.0001
CRE	1.22	0.58	**0.03**	CRE	1.47	0.57	**0.01**
MRimaging	0.77	0.30	**0.009**	MRimaging	0.88	0.35	**0.01**
**Model 3**				**Model 3**			
	Estim.	Std. err.	*p*-value		Estim.	Std. err.	*p*-value
Intercept	-5.43	1.17	*p*<0.0001	Intercept	-5.36	1.26	*p*<0.0001
CRE	1.08	0.65	0.09	CRE	1.03	0.66	0.1
MRimaging	0.03	0.40	0.9	MRimaging	0.54	0.38	0.2
MRimaging+DWI	1.06	0.32	**0.001**	MRimaging+DWI	0.62	0.26	**0.02**
**Model 4**				**Model 4**			
	Estim.	Std. err.	*p*-value		Estim.	Std. err.	*p*-value
Intercept	-7.09	2.35	0.003	Intercept	-5.08	1.48	0.0006
CRE	1.35	0.77	0.07	CRE	0.96	0.69	0.2
MRimaging	0.10	0.41	0.8	MRimaging	0.56	0.39	0.2
MRimaging+DWI	1.29	0.43	**0.003**	MRimaging+DWI	0.59	0.29	**0.04**
*δADC*	0.01	0.02	0.4	*δADC*	-0.00	0.01	0.7

### Difference in ADC between pre-treatment and response MRI (δADC)

**Fig. 3:**
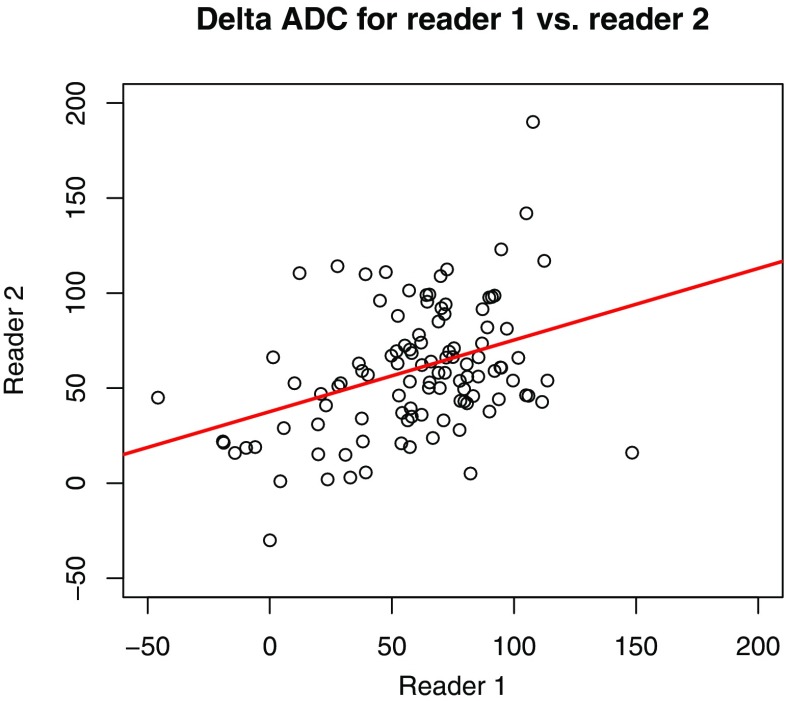
Scatter plot comparing delta ADC values for reader 1 and reader 2 shows the values were not comparable. ADC apparent diffusion coefficient

We compared δADC values for patients with a complete response and patients with residual tumour for both readers. According to the scatterplot depicting the δADC values for both readers (Fig. 3), the values of the two readers were not comparable.

**Fig. 4:**
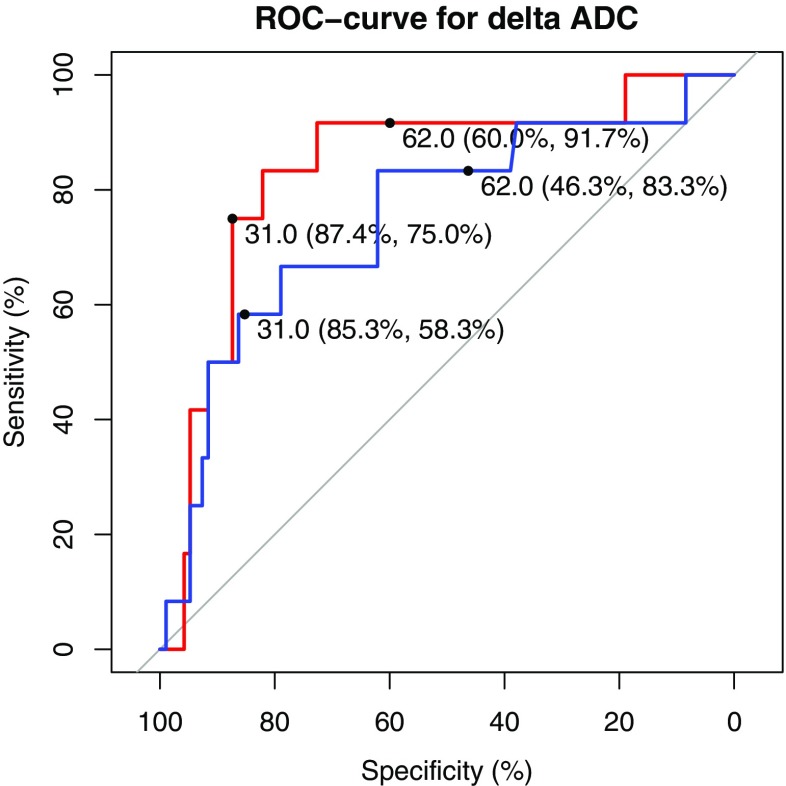
ROC analysis of change of ADC between pre- and post-treatment ADC (δADC) for reader 1 (red line) and reader 2 (blue line). Cut-offs for complete response (≥ 62 × 10^−4^) and residual tumour (≤ 31 × 10^−4^) and their corresponding sensitivities and specificities are shown

Further ROC analysis demonstrated moderate discrimination (Fig. 4): using the aforementioned cut-offs of ≥ 62 × 10^−4^ for complete response and ≤ 31 × 10^−4^ for residual tumour, the readers achieved only intermediate sensitivities and specificities.

## Discussion

In this prospective multicentre study, we found that DWI significantly increases the specificity of MR imaging in the detection of residual local tumour compared to T2W imaging alone during response evaluation of cervical carcinoma following radiotherapy. MR imaging with DWI also showed significant incremental value over CRE.

In a situation where MR imaging is appended to the treatment response evaluation, DWI information should always be added to reduce the chance of incorrectly declaring an unfavourable outcome, i.e. presumed residual tumour in cases with a complete response. This is in line with our hypothesis, since the presence of oedema hampers T2-WI, resulting in misdiagnoses of residual disease. Oedema is typically seen in the first months after radiation therapy, at the time when response evaluation is performed to decide on complementary surgery in case of residual disease. Our finding is also in line with a previous study showing that T2-WI alone resulted in up to 50% false positives [16].

Our findings are in accordance with a recent retrospective study, where a high specificity (84 %) was found using a combination of high intensity on T2-WI and high intensity on DWI [22]. However, that study differed from ours since their inclusion consisted mainly of lower stages of cervical carcinomas in a neoadjuvant setting.

To the best of our knowledge, our study is the first to compare CRE with combined T2-WI and DWI for early response assessment after radiation for cervical carcinoma. One study evaluating CRE versus conventional MR imaging in the response assessment after radiation therapy reported that MRI scored significantly worse than CRE, mainly in terms of specificity [16]. However, as that study did not incorporate DWI, it is difficult to compare their results with ours. With the current protocol, we found that MR imaging with DWI significantly improves work-up compared to CRE. It is unlikely that there was bias from the same clinician doing the clinical assessment as doing the CRE, as these assessments were done a year apart.

One could argue that as a result of current optimised radiation therapy schemes, including MR imaging or CT-guided brachytherapy, the percentage of patients with local residual tumour after radiotherapy treatment for cervical cancer is too low to justify further protocols such as MR imaging. Distant recurrence probably has more impact on survival. However, it remains a fact that only local residual tumour can potentially be cured, for instance with salvage surgery. Despite the low prevalence of local residual tumour without distant metastases, it is therefore imperative that the patient with residual tumour is recognised soon after treatment while salvage is still an option.

Logistic regression analysis showed that if both CRE and MR imaging with DWI are used, only the latter has a significant impact on identifying local residual tumour. A first option would be to replace all CRE by MR imaging with DWI. Although theoretically our data suggest that this would be an acceptable alternative, in clinical practice clinicians would still prefer to examine the patient during the first response evaluation, since it is inexpensive. Moreover, very small superficial and mucosal based residual tumours will probably remain undetected with MR imaging. A second option could be to use MR imaging with DWI in case CRE is difficult to perform or when the patient is uncooperative. A third option is to perform both CRE and MR imaging during the first response evaluation in all cases. The last two options have four clear advantages. First, the MR images can be reviewed at a later time point and can be discussed with colleagues, which is impossible with CRE. CRE also depends on the experience of the physician. A second advantage is that these initial MRI data are available during follow-up, particularly in cases in which new (clinical) problems and queries arise. The third advantage of using MR imaging is to guide eventual biopsy more precisely in the deeper cervical regions that are more difficult to evaluate with CRE. Finally, in the case of proven local residual tumour, MR imaging is usually better than CRE in identifying local parametrial or pelvic wall invasion, which could make salvage hysterectomy unfeasible, as was shown in an earlier meta-analysis by our study group [24].

A final option is to add MR imaging depending on the probability of local residual tumour and distal tumour recurrence. Higher stages of cervical carcinoma, especially those with possible lymphadenopathy outside the radiation field, would have less benefit of salvage surgery, making MRI with DWI less useful.

Quantitative evaluation of the ADC post-therapy versus pre-therapy was less helpful in our study, in contrast to findings reported in an earlier systematic review [21]. The overall absolute cut-offs found for residual tumour versus absence thereof were not reproduced in our multicentre setting. The use of different MR systems may have possibly played a role in our varied results. Indeed, a previous study has shown that absolute ADC values can be vendor-specific, depending on the region scanned in the upper abdomen [25]. As far as we know, the reproducibility of ADC values in the cervical regions across different scanners has not been fully studied. However, in terms of our findings, it is debatable whether the use of different vendors explains the differences, since only two protocols were used, with very similar sequence details and *b* values. On the basis of our findings, we conclude that measurement of ADC differences is time-consuming and is not particularly helpful, even more so because our logistic regression model showed no influence of these data on the final diagnosis of local residual tumour for either radiologist reader.

There are some drawbacks to our study. The inter-reader agreement was only moderate to fair for MR imaging evaluation, which might suggest that the results are not fully reproducible. This could be an important obstacle to further implementation of this technique. However, the readers took different approaches to image evaluation despite having received the same predetermined interpretation criteria as mentioned earlier. Reader 1 was more cautious and evaluated indeterminate lesions more often as residual tumour, thereby increasing sensitivity, while reader 2 used more strict criteria for diagnosing residual tumour, i.e. he evaluated indeterminate lesions more often as a complete response. Furthermore, reader 1 had more experience with DWI in the pelvis (10 years versus 6 years), which could also explain his higher sensitivity (although not significantly higher). Further studies should be performed to establish the reproducibility of DWI for identifying residual cervical cancer post radiotherapy.

The results concerning sensitivity were not significantly different across protocols. As a result of the low prevalence of residual disease, our study would have required over 240 patients to allow conclusions about sensitivity. For reasons of feasibility we powered the study to demonstrate an increase in specificity by adding DWI to the T2-weighted imaging.

According to the most recent guidelines, treatment evaluation should be done around 3 months after the end of treatment [15]. We decided to perform the MRI some weeks earlier, mainly because we wanted to diagnose residual tumour as soon as possible, in order to enhance the possibilities of local salvage. Too soon after treatment though, the interpretation could be hampered by oedema. However, we prioritised early detection, as the use of DWI for identifying tumour is not compromised by local oedema.

In conclusion, the addition of DWI to the standard MRI protocol significantly improves the value of MR imaging. Furthermore, MR imaging with DWI has significant incremental value relative to CRE in finding residual cervical tumour after radiation therapy, and could possibly replace the latter in situations where CRE is less practical. ADC has no incremental diagnostic value.

## Abbreviations

ADC: Apparent diffusion coefficient
δADC: Change in apparent diffusion coefficient
CRE: Clinical response examination
DWI: Diffusion-weighted imaging
MR: Magnetic resonance
T2-WI: T2-weighted MR images
